# Intellectual property issues for open science practices in genomic-related health research and innovation in Africa

**DOI:** 10.1093/jlb/lsae026

**Published:** 2024-12-17

**Authors:** Aishatu Eleojo Adaji, Lukman Adebisi Abdulrauf

**Affiliations:** Department of Public and International Law, Osun State University, Osogbo, Nigeria; School of Law, University of KwaZulu-Natal, Durban, South Africa; School of Law, University of KwaZulu-Natal, Durban, South Africa; Department of Public Law, University of Ilorin, Ilorin, Nigeria

**Keywords:** Africa, genomics, health, intellectual property rights, open science, research and innovation

## Abstract

This paper considers the applicability and implications of intellectual property rights (IPRs) for open science practices in the context of genomic-related health research and innovation in Africa. The first part provides a brief background of the gaps in genomics and health research in Africa, highlighting the possible role of open science in facilitating collaborative research to address the peculiar health needs of the continent. The second part examines intellectual property protection in genomic-related health research and innovation in Africa, outlining some of the existing legal instruments and policies guiding the application of IPRs, focusing on patents and copyrights. Thereafter, the paper examined how intellectual property standards could impact the adoption of open science in genomics health research in Africa. In doing this, the paper considers the role they could play as enablers of open science practices in genomics health research and innovation and the potential challenges they pose. The paper concludes with recommendations regarding aspects of the intellectual property policies and legal frameworks in Africa that could be calibrated to overcome potential challenges and, thereby, stimulate the adoption of an open science model and promote open, collaborative genomics health research and innovation in the continent.

## I. BACKGROUND

Currently, Africa largely depends on others for medicines and health technologies due to the relatively low capacity for research and development (R&D) and local production in the continent.^1^ This dependency raises several issues that impact the accessibility and affordability of healthcare in African countries, and these include the high prices of imported medicines and other health products resulting from import costs, such as freight, duties, and value-added tax, and a lack of effective pricing policies and regulations.^2^ Another issue is its potential to undermine Africa’s response to health crises and pandemics, such as the coronavirus disease pandemic (Covid-19), as countries on whom Africa depends could engage in stockpiling of medicines and other health products and restrict exports for their own advantage.^3^ Furthermore, while adverse drug reactions (ADRs) in Africa are attributed to various factors such as drug dose and frequency, a significant proportion is considered to be linked to variations in the human genome.^4^ Because much of the genetic research by pharmaceutical companies and medical practices around the world is informed by genomic data from other continents, particularly America, Europe, and Asia, as opposed to Africa, it implicates the efficacy of imported medicines or health products as they were developed for and clinically tested on a different population.^5^ All of these issues associated with Africa’s continuing dependency are compounded by low investments in R&D for diseases mainly affecting Africa, such as neglected tropical diseases (NTDs).

Key to addressing the forgoing issues and thereby improving public healthcare across Africa is fostering African-led research and innovation that leverage genetic diversity in the continent through the adoption of open science. Open science encompasses diverse open movements, including open access to publications, open data, free and open-source software, open collaboration, open peer review, and open educational resources.^6^ Thus, it provides a framework that could enable timely and equitable access to data, tools, and other research resources or outputs for local researchers in human genomics research and health research in general. Significantly, it engenders collaborative research and innovation within and beyond the traditional research community, bringing together governments, researchers and research institutions, philanthropic organizations, and pharmaceutical companies, among others. Through these, open science is believed to potentially reduce the existing inequalities in science, technology, and innovation and accelerate progress toward implementing the 2030 Agenda and achieving the Sustainable Development Goals and beyond, particularly in Africa.^7^ With specific regard to human genomics research in Africa, it could accelerate the scientific process, bridge the genomic data gap and bring about early scientific discoveries and technological breakthroughs targeted at addressing pressing health challenges in the continent, including chronic diseases such as sickle cell disease, diabetes, and hypertension and infectious and recurring diseases such as the Human Immunodeficiency Virus, tuberculosis, yellow fever, Ebola virus disease, Lassa fever, cholera, and malaria, NTDs, and antimicrobial resistance.

In line with international standards, such as the United Nations Educational, Scientific and Cultural Organization (UNESCO) Recommendation on Open Science 2021, open science in the context of genomic health research in Africa must be as open as possible and closed as necessary.^8^ In other words, in adopting open science in genomic health research, regulators, researchers, and other actors in Africa must consider the need to strike a balance between openness in research, development, and innovation, on the one hand, and issues like intellectual property rights (IPRs), privacy and data protection, security, on the other hand. This paper concentrates on issues concerning IPRs. The main aim is to examine how the existing intellectual property (IP) laws in Africa could shape the adoption of open science in health and genomics research by researchers in the continent. Specifically, it analyzes the challenges IPRs could pose to applying open science to genomic-related health research in Africa. The main thesis of this paper is that Africa needs to adopt non-exclusive approaches to IPRs to properly seize the potential afforded by open science to advance genomic-related health research and innovation to ensure public health in the continent.

## II. PROTECTION OF IPRS IN GENOMIC-RELATED HEALTH RESEARCH AND INNOVATION IN AFRICA

### II.A. IP Frameworks in Africa

An increasing number of African countries have in place or are in the process of designing national IP policies, strategies, and/or development plans. While IP policies are understood as overarching frameworks that set out aims and objectives for government programs directed at issues affected by and affecting IP, IP development plans are comprehensive documents consisting of a country’s policy direction, strategy, and plans on IP.^9^ In other words, IP development plans translate IP policies into concrete strategies and action plans. Countries that have formulated their own national intellectual property policy include Nigeria, Zambia, South Africa, the Gambia, Uganda, and Rwanda.^10^ Those that have adopted intellectual property development plans include Senegal, Liberia, and Mauritius.^11^ The likes of Ghana and Botswana have both intellectual property policies and development plans in place.^12^ These various intellectual property instruments, most of which are recent developments, appear to implicate issues such as healthcare, scientific research, and technological innovation, but they may have no significant effect on Africans in that they largely constitute afterthoughts. As Ncube noted, legislation should ideally be ‘informed by a thoroughly researched policy that has been comprehensively consulted upon’. However, many African countries have had intellectual property legislations imposed on them during colonial rule and, as discussed shortly, have had to amend their laws to comply with international agreements such as the World Trade Organization (WTO) Trade-Related Aspects of Intellectual Property Rights (TRIPS) Agreement, ‘without the benefit of meaningful national policy formulation’, so their reality is far from the ideal.^13^

National legislation formally protects IPRs, such as patents and copyrights, in various African countries. In Nigeria, the Patents and Designs Act 1970 and the Copyright Act 2022 protect patents and copyrights, respectively. South Africa’s Patents Act, 1978 and Copyright Act, 1978 similarly confer patents and copyrights on inventions and creations such as literary and artistic works. Meanwhile, the Industrial Property Act of 2001 and the Copyright Act of 2001 protect patents and copyrights in Kenya. Like Nigeria, South Africa, Kenya, and other African countries, Ethiopia and Ghana have intellectual property legislation protecting patents and copyrights within national boundaries.^14^ The African Intellectual Property Organization (OAPI) establishes a centralized intellectual property system for member countries, including Burkina Faso, Cameroon, Congo, Ivory Coast, and Guinea Bissau. Under this system, an Industrial Property Office common to all member countries is established to implement and apply a uniform law known as the Agreement Relating to the Creation of an African Intellectual Property Organization (Bangui Agreement) 1977 (revised in 1999 and 2015). In other words, member countries of the OAPI do not have independent national patent laws. Rather, the Bangui Agreement serves as the national law for each.^15^ As regards copyright protection, the national copyright legislation of member countries applies but only to the extent that they are not contrary to Annex VII of the Revised Bangui Agreement, 2015, dealing with literary and artistic works.^16^ In contrast, ARIPO (African Regional Intellectual Property Organization), which consists of 22 member countries, including Botswana, Gambia, Ghana, Kenya, Rwanda, Uganda, and Zimbabwe, plays mainly a facilitative role in the administration and implementation of intellectual property regimes among its member states. Patents may be granted by ARIPO under the provisions of the Harare Protocol on Patents and Industrial Designs 1982 (as amended). However, it may have no effect under the national law of a member country, which, for instance, requires that every application be filed initially with its national industrial property office or filed directly with another authority upon prior authorization unless the requirements are met.^17^ Copyright protection is subject to the national laws of its members.

In addition to the above, the intellectual property policy and legislative trends among African countries continue to be influenced by other regional, continental, and international institutions, policies, and legal instruments. Through their various policies, the regional economic communities (RECs) such as the Southern Africa Development Community, the Common Market for Eastern and Southern Africa, the Economic Community of West African States (ECOWAS), and the East African Community are seeking to integrate and harmonize the diverging intellectual property regimes at their respective regions and ensure cooperation among member countries, as it is increasingly believed that enhancing the protection of IPRs could facilitate Africa’s competitiveness in the global economy and international trade, among other things.^18^ Ogbodo highlighted the divergence of intellectual property regimes in the various economic regions, particularly ECOWAS, as reflected in the relationship between its member countries and the two main regional organizations regulating IPRs in Africa—ARIPO and OAPI.^19^ While being members of ECOWAS, the Gambia and Ghana are also members of ARIPO, with Senegal and Ivory Coast being OAPI members. Yet, as discussed above, ARIPO and OAPI each established distinct regional intellectual property systems. Nigeria and Cape Verde, also ECOWAS members, belong neither to ARIPO nor OAPI.

Recent drives by the regional economic communities to establish coherent and harmonized intellectual property regimes at the regional level are taking place within the framework of the AU Agreement Establishing the African Continental Free Trade Area (AfCFTA) 2018. Per Article 7 of the Agreement, the broader goal is to develop an AfCFTA Protocol on IPRs to govern IPRs at the continental level. There are other continental instruments with similar implications, including the Statute of the Pan African Intellectual Property Organization (PAIPO) 2016, which seeks to establish harmonized intellectual property standards that accommodate the needs of the AU and RECs as well as ARIPO and OAPI while ensuring that the regional treaties and national legislation also accord with such standards. Significantly, it recognizes ARIPO and OAPI as building blocks for establishing a PAIPO, which shall be responsible for all issues relating to intellectual property at the continental level. These continental instruments are critical to the adoption of open science in Africa as they promote the ideals of Pan-Africanism while seeking a pragmatic approach to establishing and implementing intellectual property standards that reflect the realities and address specific intellectual property issues unique to Africa, including access to medicine and other health technology. However, it remains to be seen whether they could make a substantial difference, especially as they do not set out substantive provisions for granting and protecting IPRs. Rather, they are mainly policy statements. Regardless, in this paper, they serve as points of reference for aligning intellectual property rules with the principles of open science in the context of genomics health research and innovation in Africa.

International instruments such as the WTO Agreement on TRIPS Agreement 1994 (as modified), the WIPO Patent Cooperation Treaty (PCT) 1970 (as modified), and the WIPO Copyright Treaty (WCT) 1996 continue to substantially influence African countries, the majority of whom are members of both the WTO and WIPO. This is because they standardize the substantive and procedural rules concerning the implementation and enforcement of IPRs. Particularly, the TRIPS Agreement has far-reaching implications because it is the most comprehensive and authoritative international instrument, setting the minimum standard for protecting IPRs. Making the WTO’s dispute settlement procedures an integral part of the agreement significantly provides for the use of commercial retaliations originally conceived to resolve trade issues. This ensures that member states of the WTO, a significant per cent of whom are African countries, are TRIPS compliant irrespective of whether the international IP standards in the TRIPS Agreement conflict with their developmental goals and aspirations. In this vein, intellectual property legal instruments are reviewed or designed at various levels in Africa to bring them in conformity with international standards. For instance, to correspond with international standards, the OAPI Bangui Agreement of 1977 was amended in 2015, the ARIPO’s Protocol on Patents and Industrial Designs 1982 has also been subject to a series of amendments, and a new Nigerian Copyright Act was enacted in 2022, repealing the Copyright Act of 1988. Similarly, although the RECs provide intellectual property policy models and guidelines for countries to formulate their intellectual property policies and legislation, they often partly affirm that member countries commit to abide by international intellectual property standards.

**Figure 1:**
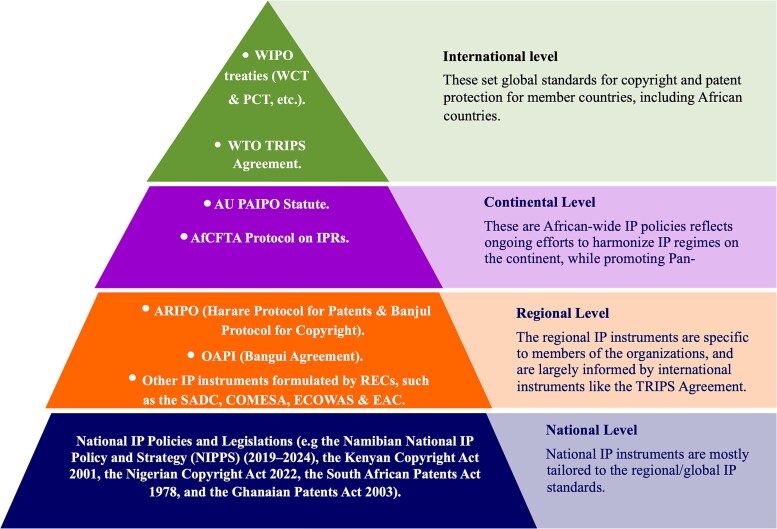
Key legal instruments influencing copyright and patent protection in Africa.

However, the current IP standards, as embodied in international instruments such as the TRIPS Agreement, 1994 (as amended), have been a subject of continued controversy, particularly as regards access to medicines and other health technologies in Africa and other developing countries. Efforts to counter the perceived shortcomings of the existing globalized IP system, which included restricted access to technological innovation, led to the emergence of open science and other open movements. However, open science potentially conflicts with the existing IP standards. Thus, in the next section, this paper analyzes how the current IP frameworks could challenge the adoption of open science in human genetic research and innovation in Africa. Figure 1 summarizes the key legal instruments influencing copyright and patent protection in Africa referenced in this paper.

### II.B. Scope of IP Protection in the Context of Genomic-related Health Research and Innovation

A broad range of intellectual creations, such as inventions, data, and literary, scientific, and artistic works, among others, are protected under various intellectual property instruments in Africa. These existing IP instruments are generally conceived on the premise that the grant of exclusive or proprietary rights, particularly in the scientific field, would engender research and development, creativity, inventiveness, and technology transfers, enabling wealth creation for individuals and companies while facilitating public access to intellectual creations. In other words, intellectual property systems seek to achieve the twin objectives of safeguarding the private ownership rights of researchers, inventors, or creators, among others, on the one hand, while promoting public interests in accessing and using the intellectual creations on the other hand.^20^ The exclusive rights, collectively known as IPRs, are not absolute in that they are restricted in various ways under the existing intellectual property instruments, including by duration, compulsory licensing, and research use exemption, to strike a balance between private and public interests. As concluded in the latter part of this paper, these exemptions could be instrumental in facilitating Africa’s adoption of open science in genomics health research and innovation.

Although the term IPR is often used interchangeably with intellectual property, it is worth stating that in this paper, the term intellectual property is specifically used to connote various intangible products of the human intellect, their physical manifestations, or expression of which legal protection and rights are being granted to enable the owners to exercise exclusive control and exploitation. Intellectual property in health and human genomics may manifest in isolated human DNA sequences or genetic databases, biomedical research tools, computer programs, diagnostic tools and processes, drugs, and other gene-based products and processes or even be expressed as a book or journal publication. For these, various forms of IPRs are obtainable under the existing legal instruments in Africa, including patents, copyrights, trademarks, trade secrets, and designs. Among other things, these rights, when granted, enable the owners to recuperate the costs of researching, creating, or inventing and make substantial financial gains from exploiting the intellectual properties. Also, the owner may claim pecuniary compensation for infringement if anyone exploits these rights without authorization. In addition, some legal instruments recognize the moral rights of authors over their copyrighted works. Moral rights often confer authors the right to claim authorship and ensure their works are treated with integrity and not subject to derogatory actions.

While the existing legal provisions on patenting highlighted earlier may be at variance in certain regards, it is worth noting that, in relation to genomic health research, patent rights have been expanded over the years to cover products and processes developed from or relating to genetic materials and information, including messenger ribonucleic acid (mRNA), mRNA vaccines and therapeutics, genetic tests, predictive biomarkers, and biomedical engineering tools and techniques, but with the assertion of patent claims over DNA sequences isolated from human body remaining controversial in Africa, as in other jurisdictions.^21^ Generally, whether gene-based or not, inventions must meet the patentability standard tests of novelty or newness, non-obviousness or inventive step, and industrial application to be protectable. However, even when a product or process can meet the patentability standard tests, it may not be considered a patent-eligible subject matter of patent due to the exemptions as may be stipulated under relevant legal instruments. For instance, a patent may not be claimed over discoveries in some jurisdictions.^22^ In these regards, critics of gene patents have argued that genes are naturally occurring substances and that ‘while much intellectual effort may have gone into discovering them within the DNA sequence, discovery is not the same as invention’.^23^ It is also argued that ‘with the completion of the Human Genome Project in 2003, all of the human gene sequences were in the public domain’ and, therefore, are novelty-defeating prior art.^24^ It is further pointed out that ‘discovering the location of a gene never rose above the bar for being non-obvious’, as it is believed that by the late 1990s, the practice had become commonplace.^25^

The criticisms stem from gene patents’ potential to inhibit biomedical research and clinical applications such as gene testing. This is because when a patent is granted in relation to a particular DNA sequence, the holder dictates how it can be used during the patent’s duration, including for research and commercial genetic testing.^26^ It often results in only the holder ‘having sole ownership of genetic testing for patented genes’.^27^ Gene patents create barriers to innovation in genetic testing as the patent claim over the actual DNA sequence to be tested grants only the holder the right to sequence that DNA during the patent’s duration.^28^ Despite these challenges associated with gene patenting, it remains in practice around the world. In the United Kingdom (UK) and countries under the European Union (EU), isolated DNA sequences are patent-eligible subject matters and are capable of meeting the criteria of newness, inventiveness, and industrial application.^29^ In the United States (US), it has long been established by the US Supreme Court’s 2013 ruling in *Association for Molecular Pathology v. Myriad Genetics, Inc*., that isolated DNA sequences in their natural forms (unmodified) are not patentable.^30^ However, modified, laboratory-created, or synthetic DNA sequences are patentable. Although the approaches vary, it is not clear where Africa stands, even though it has been suggested that South Africa’s approach will likely align with the EU’s.^31^

While patents may have the widest impact on health and human genomics research, development, and innovation by researchers in Africa, copyright also impacts access to genetic knowledge and data, particularly for researchers in Africa. Copyright protection applies to various modes of scientific communications, including figures, images and graphics, dataset compilation, scientific publications like journals and articles, conferences, and seminar presentations. Digital technologies are increasingly used for biomedical research and many other contexts. The software programs are commonly considered copyright subject matter under the various copyright instruments on the African continent. They have the same status as literary works in some jurisdictions and are categorized as *sui generis* works, different from literary works in others.^32^ Unlike patents, the subsistence of copyright does not depend on formalities, such as registration. Among others, copyright comprises the rights to reproduce (make copies) and publish (communicate and make copies available to the public).^33^ Authors are automatically vested with the copyright ownership, except when the law stipulates otherwise. However, copyright works, such as publications and data, are often behind paywalls as authors routinely transfer their exclusive economic rights to commercial publishers under existing publishing models.^34^ This deeply impacts access to, use, reuse, and sharing of data and other scientific resources for African research and innovation.

**Figure 2:**
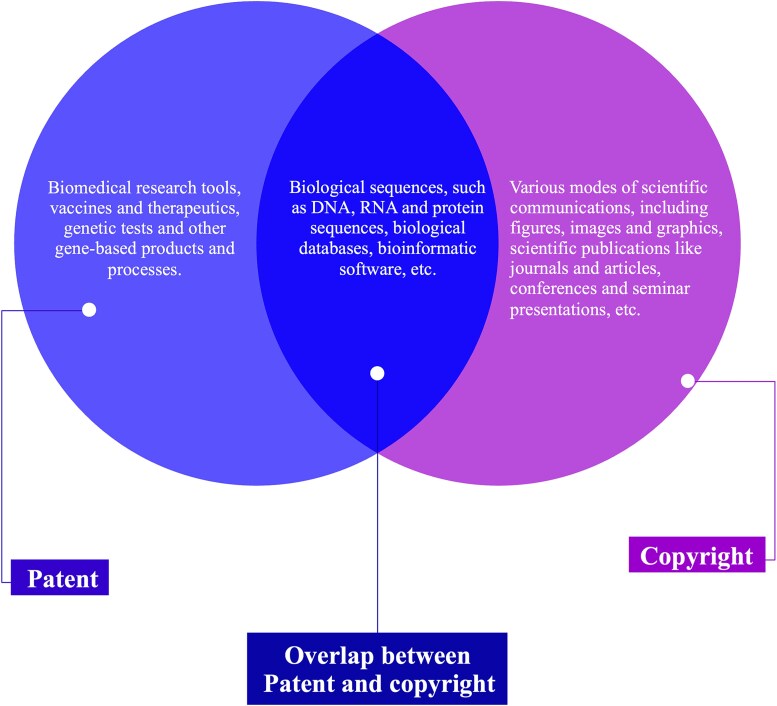
Patent and copyright protection in genomic-related health research and innovation.

The same research input/output may be subject to different rights in genomics health research. For instance, patents and copyrights may be reflected in genetic sequences/databases, as shown in Fig. 2. Also, these IPRs are distinct from other rights or interests in genetic resources, such as the data privacy rights of the person who provided the genetic resources from which inventors/authors developed their patentable or copyrightable materials.^35^ Nevertheless, it is worth noting that there is an increasing considerable overlap between the creation or exercise of IPRs in human genomics and other spheres of health research on the one hand and the data privacy rights of the human research participants on the other hand, which could pose additional obstacles to data access and reuse by researchers.

## III. INTELLECTUAL PROPERTY ISSUES AND CHALLENGES IN OPEN SCIENCE

The notion of open science is not new to the academic and scientific communities because the sharing and dissemination of research results represent a well-known tradition among scientific researchers. This is guided by the belief that science is cumulative and sequential, so sharing and access to accumulated scientific information and data is a prerequisite for follow-on research and the general progress of science and technology.^36^ In line with the traditional scientific norms of communism, universalism, disinterestedness, and organized skepticism, the central tenets of open science are: transparency, scrutiny, critique, and reproducibility; equality of opportunities; responsibility, respect, and accountability; collaboration, participation, and inclusion; flexibility; and sustainability.^37^ On this basis, open science potentially furthers the rights of Africans to health and ‘share in scientific advancement and its benefit’ as enshrined in Articles 25.1 and 27.1 of the Universal Declaration of Human Rights (UDHR) 1948 and other human rights instruments. It also promotes the right to scientific freedom, specifically in Article 15(3) of the International Covenant on Economic, Social and Cultural Rights (ICESCR) and other national instruments, such as section 16(1)(d) of the South African Constitution.^38^

In addition to international standard-setting instruments, such as the UNESCO Recommendation on Open Science 2021, there are various national policy instruments in Africa suggestive of governments’ commitments to open science, including Ethiopia’s National Open Access Policy 2019, Nigeria’s Revised National Science, Technology and Innovation Policy 2022, and South Africa’s Draft National Open Science Policy 2022.^39^ These open science instruments recognize the rights of scientists to claim IPRs over their research outputs in line with Article 15(1)(c) of the International Covenant on Economic, Cultural, and Social Rights and existing legal frameworks, some of which were referenced in the previous section. However, the exclusivity and individualistic nature of rights under the existing intellectual property system inherently present challenges to attaining Africa’s open science goals in genomics health research. These arise in two broad contexts. First, scientific researchers, innovators, and open science actors may face challenges regarding using, reusing, and sharing resources for research purposes, where such resources are subjects of IPRs. Secondly, they will also be faced with uncertainties regarding the status of the research outputs. These points are discussed below.

### III.A. Implications of IPRs on Research Inputs

In the context of open science, even though protected, existing intellectual property works, such as scientific knowledge, data, source codes, methods, and processes, can be incorporated into research and innovation processes. However, this is subject to the authorization of the right holders. Relying on the distinct features of open licensing, IPRs may be exercised to enable the shared and collective use of IP by researchers and open science actors and engender open collaborative research and development, particularly in genomics health research in Africa. As a strategy, open science initiatives or policies may encourage right holders to assign or license their IPRs to a project to clearly define the rights granted to users over the intellectual property contributed and for effective enforcement or defense of the IPRs against unpermitted use.^40^ With specific regard to scientific and scholarly publications, it is also becoming common for open access policies of institutions and funders to encourage or mandate their researchers to retain rather than completely transfer or exclusively license their copyrights to publishers to ensure that research results can be made open and publicly accessible under open licenses.^41^ Examples of open licenses include the Creative Commons licenses for copyrighted works, in general, and the GNU licenses and Open Source Initiative (OSI)–approved licenses for copyrighted software and related documentation.^42^ On this basis, it could be argued that IPRs are not antithetical to open science.^43^

Despite the above, the underlying philosophical bases of IPRs and open science, as reflected so far, are fundamentally different. While open science is inclusionary, IPRs remain primarily instruments of exclusion. Thus, except for resources in the public domain, the applicability and sustainability of open science also hinge on the voluntariness of researchers, innovators, or right holders in sharing and allowing the reuse, modification, and distribution of their intellectual property. However, there is no guarantee that holders of IPRs will exercise their proprietary rights in such a way as to permit unrestricted access, use, reuse, and sharing of their intellectual properties for open-science research and innovation. In other words, there is a high probability that existing intellectual property systems could constrain open science adoption in genomics health research in Africa.

Specifically, IPRs are used to keep others out in critical aspects such as health and medical biotechnology. It is well established that patent holders are often reluctant to grant licenses out of the desire to prevent competition and protect monopoly pricing.^44^ This puts platform technologies, methods, and associated genetic databases critical to genomics health research out of reach of biomedical scientists, constraining follow-on research, improvement, and adaptation of existing technology in general and in the context of open science. Similarly, even though open-access publishing models are emerging, publishers of closed-access journals and other forms of publications continue to use copyrights to impose high subscription rates to maximize profit.^45^ This obstructs access to scientific literature and the free flow or dissemination of scientific information and data relevant to advancing open science in Africa’s genomics health research and innovation.^46^ It is worth noting that while researchers may opt for any of the existing open-access publishing models and thereby make their works available to anyone without copyright restrictions, they may be required to pay a significant amount as article processing charges that will be prohibitive for those in Africa, due to inadequate funding. Furthermore, copyright retention strategies in open-access policies could create problems for researchers if the publisher rejects the terms of the license and requires assignment or an exclusive license. All of these suggest a need to expand the open science discourse to practical mechanisms that could incentivize researchers and innovators to adopt open practices in genomics health research in the African context.

### III.B. Implications of IPRs on the Research Outputs

Another issue between open science and IPRs in the context of genomics health research and innovation in Africa arises in relation to the outcomes of the research and innovation process. Open science, as noted, aims to make scientific resources, including research outputs openly available, accessible, reusable, adaptable, and shareable by everyone. This is attained by open licensing or dedication to the public domain, where appropriate.^47^ Like a dedication to the public domain, an open license may grant licensees (users, researchers, or biomedical scientists in the context of genomics health research) unrestricted rights, including to use, reuse, copy, modify, adapt, or improve and share open-science research products, both resulting in the creation of commons. However, open licenses may be non-copylefted (non-reciprocal) or copylefted (reciprocal or ‘grant back’ licenses), imposing additional terms on licensees. For instance, as is common with open-source software movement, a copyleft license may require licensees to share their improvements to a licensed work or invention with other users on similarly unrestricted.^48^ Obligations as such do not arise with regard to works or outputs placed in the public domain. The suitability of either of the approaches to open science outputs—dedication to the public domain and copyleft or non-copyleft licensing—may be judged based on the goal of the open project and the nature of the output or stage in the development process, among other possible criteria.

Significantly, as again exemplified by the open-source software movement, open licenses rely on copyright or other IPRs to enforce their terms. In this respect, it is worth noting that paragraph 16(b) of the 2022 UNESCO Open Science Declaration particularly suggests protecting the intellectual creation of open science methods, products, and data. Some contributors to the open source/open science discourse are more inclined to the dedication of open science outputs to the public domain instead of openly licensing them due to the need for an underlying property right for efficient licensing.^49^ However, while the public domain approach may serve commercial interests, it may not always be in the public interest. Users may obtain proprietary rights over improvements based on previous research outputs openly shared in the public domain and assert their rights against other interested users, denying them the same freedom they enjoyed in accessing and using the resources underlying their improvements. This could disincentivize others from continuing to contribute or invest in the open science process.

Rai argued that right holders who benefited from the public domain or non-copyleft licenses ‘may feel a norm-based obligation to contribute their improvements back’, even as they are not under any legal obligation to do so, but it is far from certain that they will do so or allow access under favorable terms.^50^ In light of this, this paper argues that open licensing is required as a complementary mechanism, particularly with regard to improvements that cannot be invented around or constitute research tools necessary to advance fundamental research in areas like human genomics. It provides options for the licensors ‘to reserve specific rights related to commercial use and the sharing of modifications, and requiring that the same or similar license is applied to derivative work’.^51^ Moreover, considering the continent’s level of genomics research, it may be more beneficial to leverage the open license mechanism to ensure grant-backs to sustain open science practices in genomics health research and innovation in Africa while preventing unfair and/or inequitable exploitation of open science resources.

IPRs provide a legitimate basis for enforcing open license terms.^52^ By inverting or repurposing existing intellectual property rules, open licensing schemes are used to maintain open science practices, control predatory behaviors, and guide against private appropriation of open science outcomes by a few to exclude all other users.^53^ This makes open science more broadly accessible and available, allowing people ‘to build on it and multiply its impact—including by sharing helpful improvements and modifications’.^54^ Underlining the importance of IPRs in this regard, van Overwalle notes that ‘the well-functioning of open source is dependent on “credible commitment”’.^55^ Credible commitment in the open science context requires that the intellectual products be protected by IPRs or ‘other proprietary rights and distributed on terms that are perceived to be legally enforceable’.^56^ As discussed above, genomics-based products are capable of intellectual property protection. However, besides the high costs associated with prosecuting and enforcing IPRs, there is a fundamental question of whether IPRs, particularly copyrights and patents, are accruable to genomics in the context of open science or whether the existing intellectual property standards are amenable to open science practices in genomics health research. It further raises the question of who the rights are accruable to or whether the existing intellectual property standards in Africa can suitably capture the equal treatment of all the participants under the open science model. These are discussed in relation to genomics health research.

Generally, the open, collaborative, and inclusive characteristics of open science foster a research and innovation process where all contributions are valued, and researchers/actors are recognized and allowed to enjoy equal entitlements to the benefits of open science regardless of the quality or quantity of their contributions.^57^ In other words, open science is not tied to a particular person or entity; rather, it relies on the intellectual contributions of a potentially diverse and unlimited number of contributors. Accordingly, the ensuing outputs, including genomic datasets/databases in the context of genomics, derive from and build on the integration of collective and accumulated intellectual efforts or resources and, to that extent, engendering a non-exclusive or inclusive entitlement to the outputs among the contributors as well as users. However, as discussed below, this open collaborative model of research, where no single person may be identified as the sole creator/inventor of a work/invention, is challenged by the traditional notion of copyrightability and patentability obtainable under the existing intellectual property system, impugning the enforceability of the inclusive entitlement of contributors and users.^58^

#### III.B.1. Copyright protection

Works of plural authorship can be broadly classified into collective and joint works. Their copyrightability generally hinges on various requirements, including their existence in a static form to which authorship can be attributed.^59^ In other words, a work, whether as a joint or collective one, comes into existence when it is expressed in a form that is ‘sufficiently stable to permit it to be perceived, reproduced or otherwise communicated for a period of more than transitory duration either by human sense(s) or with the help of a machine’.^60^ However, because of the open, collaborative, and inclusive nature of open science, it fosters a highly dynamic and sequential creation process in which a series of intellectual contributions may continually and rapidly be absorbed for an undefined period of time.^61^ The question may be whether the successive developments were sufficiently stable or merely evolutionary. Could the successive developments be categorized as a collective work, or do they merely constitute stages in the evolution of a joint work?

In the French case of *Thomas et SARL Ready Soft v. SARL Codat Informatique et Mattern*, Mendis noted that rather than compartmentalizing each stage of software development as new derivative creations, the Court of Appeal of Versailles, France, held the successive developments of software program to be a collective work, the individual contributions of various authors having merged in such a way that it is not possible to assign a separate right to each of their contributions.^62^ In this context, the successive versions of a software program were considered to represent stages of the software program’s evolutionary process. Article 1(v) of Annex VII to the Bangui Agreement 1977 similarly considers collective works as works ‘… in which the individual contributions of the various authors participating in its creation merge in the whole for which it was created so that it is impossible to attribute to each of them a separate right in the whole work once completed’.^63^

However, the meaning ascribed to collective works under the Bangui Agreement 1977 and the French judicial decision is most commonly used to describe works of joint authorship or jointly created works. It is more appropriately captured as such under various national copyright laws, including the South African Copyright Act 1978 (as amended) and the Ghanaian Copyright Act 2005.^64^ For instance, section 1(1)(xlvi) of the South African Copyright Act 1978 defines work of joint authorship as ‘a work produced by the collaboration of two or more authors in which the contribution of each author is not separable from the contribution of the other author or authors’. Collective works are generally compilations and databases comprising a range of contents, such as raw data, literary or artistic works, and other materials that, although independent and individually accessible, are assembled into a new unified creation.^65^ Unlike jointly created works, where the contributions of all the authors meld into a unitary whole, the constituent elements forming the collective works remain individually identifiable. Nevertheless, it is significant to note that the Senegalese copyright law adopts a slightly different approach. Following the amendments in 2008, the term collective work was jettisoned with the joint work used in a wider sense to accommodate collaborative works ‘irrespective of whether it constitutes an indivisible whole or consists of autonomously created parts’.^66^

Traditionally, different rules apply regarding the authorship and ownership of collective and joint works. Copyright subsists in collective work by reason of the selection and arrangement of the contents.^67^ As section 108 of the Nigerian Copyright Act 2022 puts it, the author of collective work is ‘the person responsible for the selection and arrangement of the collection’. In this sense, the law presupposes that the ‘selection and arrangement’ of contributions in the creation process is subject to the single control and authority of an identifiable entity.^68^ An authoritative entity may easily be identifiable under a proprietary model, but researchers and actors in the open science context, as exemplified by open-source software projects, may operate under a less formal environment without a central command-and-control structure.^69^ While project coordinators are in charge of integrating contributions into the work and, through this process, may arguably exercise some form of minimal control, it is not clear whether it can be said that they exercise sufficient judgment and discretion in the acceptance and integration of contributions to give rise to a work over which they can claim authorship. This is because it can be argued that project coordinator(s) engaged purely in the mechanical task of incorporating contributions and they do not have ‘the ability to exercise control over the creative process or in determining the nature and form of the original expression’ that would be incorporated in the work.^70^ It would, therefore, be difficult to determine who should be vested with authorship over the intellectual outputs from open science.

Copyright in a collective work is distinct from the copyright that may subsist in the various contents constituting the collective work.^71^ Thus, while co-authors of joint works own the copyright jointly and equally, the author of the collective work has a broader ownership interest in the full work, compared to the contributors whose entitlement is limited to the constituent elements they contributed to the collective work. This undermines the principles of open science, which promotes equal opportunities to access, contribute to, and benefit from open science. In this regard, it is worth noting that the approach obtainable under Articles 23–25 of the Senegalese Copyright and Related Rights 2008 is quite distinct, as it is suggestive that even when the contributions are identifiable, contributors could enjoy equal rights in the common work. Significantly, while contributors of the data and materials incorporated in a collective work could exploit their contributions independently of the right subsisting in the collective work, it is also possible that their individual contributions may not satisfy the originality standard test even though through their combination with each other, they give rise to a copyrightable collective work.^72^

Regarding works of joint authorship, the referenced copyright laws, including the Senegalese copyright law, envisage some form of predetermined relationship among the contributors, which ‘must be in furtherance of a joint or common design’.^73^ In this sense, it also means, as joint authors, they are identifiable and, together, exercise control over the creation process and the design or form of the final copyrightable work.^74^ Yet, by making the scientific and innovation process more inclusive and accessible, open science engenders contributions from contributors who do not know each other, although, as Demil and Lecocq note, a small group of well-known actors could emerge.^75^ With contributors allowed to enter and exit the open science community at will, the membership and relationship among participants is open and undefined. While the work may meet the originality and fixation requirements, the notion of joint authorship under the existing copyright laws is not suitable for capturing the equal treatment of contributors under the open science model.

Similarly, the current notion of authorship/joint authorship also undermines the core values and guiding principles of open science, such as equality of opportunities, diversity, and inclusiveness, fostering an environment where all contributions are valued, and researchers/actors are recognized and allowed equal entitlements to the benefits of open science regardless of the quality or quantity of their contributions. The existing copyright standards referenced above generally define the author, particularly in relation to literary work, as the person who creates a work. Oyewunmi maintains that copyright law envisions a creator as ‘the person who expends effort, in terms of input of labour, judgment and skill in creating the particular expression that constitutes a work’ that qualifies for copyright protection.^76^ Thus, to qualify as joint authors, each contributor must be a creator. In other words, each contributor’s contributions must be directed toward the original expression of the final work that gives rise to copyright, although the quantity and quality of contribution need not be equal.^77^

Applying the conventional notion of authorship in the open science context would mean that persons who merely contribute ideas or make purely technical or non-expressive inputs such as typesetting or editing and fine-tuning without more will not be entitled to claim the right of joint authorship.^78^ Besides, the expressive contributions of some contributors may become obscure following successive developments in the scientific process.^79^ Consequently, this paper aligns with the views of Mendis, who pointed out that in limiting authorship only to persons who have contributed toward the expression of the work, the copyright system ‘effectively discriminates against those contributors who engage in vital tasks of editing and commentary which, although not directly contributing to the expression of an open collaborative work, are nevertheless crucial in sustaining the creation process and could serve to influence the nature and form of the common work’.^80^ The writer also argues that such discrimination ‘militates against the basic ideological norms of equality, collectiveness and sharing’, fueling the open collaborative knowledge generation and innovation process.^81^

#### III.B.2. Patent Protection

Patent laws recognize joint patenting and ownership. This is exemplified by section 24 of the Nigerian Patents and Designs Act, 1970.^82^ Specifically, subsections 1 and 5 of the said section are to the effect that two or more persons may have the right to bring an application for or be granted a patent as ‘joint applicants, joint patentees or joint owners as the case may be’. On the face of it, these provisions may seem to accommodate the collaborativeness and inclusiveness of the open science research and innovation process. However, it is worth taking note of section 2(5) of the Nigerian Patents and Designs Act 1970, a careful examination of which suggests that the form of joint inventorship contemplated within the law forecloses the right to claim joint inventorship by open science actors whose intellectual efforts are geared towards the contribution of information or ideas and deliberations on or fine-tuning of scientific/technical ideas and innovations. This is because it recognizes inventors only as contributors of ‘inventive activity’ and not those who ‘merely assisted in doing work connected with the development of an invention’, however important their contributions are to the initiation, development, and sustainability of the innovation process.^83^

As open science is collaborative and predicated on the sharing and exchange of scientific knowledge and other intellectual resources among researchers, a further issue arising in the context of patent is whether the sharing and exchange of scientific data and other intellectual resources in the scientific and innovation process can be deemed public and thereby constituting patent-defeating prior art? In other words, can open science principles hamper future applications for patent protection? Conversely, the fact that researchers and innovators engaging in open science research could apply for patent protection regarding open science outputs also raises the question of whether the novelty requirement and the duration (beginning from when a patent is filed to when it is granted) do not undermine open science research and development process?

Critics have often argued that patenting impedes the sharing of scientific knowledge and other intellectual resources.^84^ They suggest that, due to the novelty requirement, researchers and innovators are compelled to maintain some form of secrecy, even among colleagues, for the sake of future patent applications.^85^ The implication of patenting becomes of more critical importance in relation to the open science model, which is predicated on the free flow of information, data, and other intellectual resources among an even larger and wider community comprising users, researchers, innovators, and other actors. This is because the potential that the sharing and exchanging of intellectual resources may pose a challenge in patenting the ensuing invention could serve as a disincentive for researchers or innovators to engage in open scientific research. In other words, in a bid to meet the novelty standards, participants may be reluctant to share their contributions and improvements in the open science community and may only do so after the filing of a patent application. This would damage the collaborative open scientific innovation process and the advancement of science, as the novelty requirement delays or prevents public sharing of key information or intellectual resources among participants.

However, as desirable as it is to gain quick and unrestricted access to improvements, including patentable but unpatented technology, the situation is complicated by the very fact that, as noted above, patent claims and other forms of proprietary rights provide the legal basis for the enforceability and sustainability of open licensing such as the copyleft license. When research outputs over which there are no proprietary rights are distributed under open licenses, such as the copyleft type, it essentially amounts to a dedication to the public domain.^86^ Takenaka has particularly argued that disclosures pursuant to patent applications ‘are more useful than other types of voluntary disclosures because they prevent others from obtaining patents on the disclosed inventions more effectively than other disclosures and promote collaboration among innovators’. ^87^ As pointed out earlier in this paper, patents and other forms of IPRs, when obtained in the open science context, are used for inclusive purposes to facilitate open, collaborative research and innovation processes. Arguably, reducing the risk of free riders exclusively appropriating the inventions incentivizes researchers and innovators to engage in open science practices.^88^

## IV. POSSIBLE WAY FORWARD FOR AFRICA

This section offers a few thoughts on aligning IPRs with open science adoption in Africa’s genomics health research.

### IV.A. Sustaining Open Science through the AfCFTA Protocol on IPRs

The AfCFTA Protocol on IPRs is emerging as a crucial component in Africa’s intellectual property landscape. Therefore, it is a vital tool for advancing open science in genomics health research and standardizing IPRs in Africa. Among other things, the draft Protocol seeks to promote African innovation and creativity, access to knowledge, and Africa’s public health needs.^89^ It emphasized the need to facilitate ‘access to medicines, vaccines, diagnostics, therapeutics, and other healthcare essential tools’ in Africa while also setting out detailed provisions furthering the protection of public health interests during emergencies, such as epidemics and pandemics.^90^ Significantly, it seeks to integrate the open licensing mechanism, research cooperation, and other collaborative models to propel innovation and facilitate the transfer and diffusion of technology in Africa.^91^ However, it does not address the barriers that, as pointed out, the exclusivity of the current intellectual property standards poses to the open, inclusive, and collaborative nature of open science.

As the negotiations continue, with additional obligations on various issues to be set out in Annexes to the Protocol, there are still opportunities to delve deeper into the interaction between the concept of open science and IPRs. Specifically, within the Intellectual Property Protocol, AfCFTA could advocate for policies that foster a culture of open science among researchers and research institutions in Africa with regard to diseases affecting mainly Africa and other areas beneficial to the continent. In this vein, it must take into account that open science, particularly in the context of genomics health research and innovation requires a different approach to intellectual property protection. Thus, in addition to encouraging member states to take steps to raise awareness about materials in the public domain and existing open licensing schemes, AfCFTA could take an entirely original approach by promoting the codification of inclusive rights advocated by the likes of Dusollier, van Overwalle, Mendis, and Takenaka, as an alternative to the current exclusive regimes on copyright and patent.^92^ The *sui generis* inclusive rights are proposed to, among other things, accommodate the collaborative, inclusive, and dynamic nature of the open-science research process and products, as well as provide options for individual authors and inventors who want to use their copyrights and patents as inclusive rights. It gives every user a ‘legal entitlement’ over intellectual property—to use and engage in traditionally excluded acts. It is also used defensively to defeat any claim of exclusivity that could hamper the common use. Furthermore, copyleft provisions are incorporated, particularly with regard to current and future inventions that could block the use and improvement of the invention/work over which inclusive right is granted.^93^

With specific regard to ‘inclusive patent’, Takenaka envisages that it would not only guarantee the holder’s rights to use and share the invention but also serve as bargaining chips to access other components of a product or services subject to numerous patents held by different patent owners.^94^ It is also proposed that the right to use the invention would include ‘the right to request a compulsory license when the inclusive patent owner would otherwise be unable to practice the invention because of blocking patents held by others’.^95^ As patents are usually expensive to obtain and often involve delays before they are granted, Takenaka further advocates that inclusive patents be granted quickly and cheaply to encourage disclosures.^96^ Mendis similarly elucidated on the inclusive right regime in the copyright context, arguing authorship be constructed in a broader sense to include ‘an entitlement to claim authorship over a work that is held by a plurality of persons in a collective and symmetrical way without any one of those persons having the power to exclude another from claiming such authorship’.^97^ While this definition focuses on copyright, it could be extended to inventorship within the patent context. In other words, so far as a person’s contribution has been absorbed in the creation or innovation process or product, the person is recognized as co-author or co-inventor in relation to the ensuing work or invention in line with the core values and guiding principles of open science.

### IV.B. Fostering Open Science in Genomics Health Research within the Boundaries of the Existing African Intellectual Property Frameworks

The various intellectual property legal instruments incorporate exceptions to patents and copyrights that may be useful in fostering open science practices in genomics health research in Africa. This is because the exceptions permit the use of IP and other acts that are the exclusive rights of right holders without their consent. For instance, by way of fair dealing, section 20 of the Nigerian Copyright Act 2022 permits researchers to use copyrighted works, such as scientific articles and compiled databases. It also permits their use by scientific institutions where it is in the public interest. In addition, it permits

communication or making available of works and other material not subject to purchase or licensing terms to members of the public for the purpose of research or private study through dedicated terminals on the premises of publicly accessible libraries, educational establishments, museums, and archives.^98^

Unpublished works kept in libraries, museums, or other similar institutions to which the public has access may also be reproduced for research purposes or private study without the right holder’s authorization.^99^ The elaborate compulsory licensing provisions under sections 31–35 of the Nigerian Copyright Act 2022 may also benefit researchers adopting open science practices in genomics health research. Article 62 of Annex II to the Bangui Agreement, section 26(1)(a) and (h) of the Kenyan Copyright Act, sections 19, 21–22 of the Ghanaian Copyright Act, and sections 12–13 of the South African Copyright Act also provides for permitted uses without the consent of right holders. Concerning patents, some national laws do not contain express provisions for research use exceptions besides Kenya, but the compulsory licensing and public interest provisions on health could provide the context within which researchers may use patented inventions and technical information disclosed to meet the enablement and written description requirements for open science–based genomics health research in Africa.^100^

It is worth noting that the exceptions or permitted uses are, in most cases, restricted, for instance, to non-commercial and private uses, specific beneficiaries (eg government, research institutions, or research persons), confined to reproduction rights as opposed to publication and distribution, and, where distribution is permitted, it may be restricted to the country’s domestic market. Also, they are treated differently within the national laws of the various African countries, with some countries not providing for research exceptions under their patent laws, as noted above. Furthermore, it is well established that the fear of retaliation and the bilateral/regional free trade agreements between the developed North and African countries often limit the use of mechanisms such as compulsory licensing in the continent.^101^ For instance, South Africa’s enactment of the Medicine Act 1997 in an attempt to address its public health challenges by employing the use of compulsory licensing and other related mechanisms led to the US placing South Africa on its ‘Special 301’ Watch List amidst threats of trade sanctions.^102^ Multinational pharmaceutical companies also instituted legal action against the South African government, arguing, among other things, that the 1997 Medicine Act was inconsistent with the TRIPS Agreement, although the suit was subsequently withdrawn.^103^ These various issues surrounding the existing patent and copyright exceptions create legal uncertainties for researchers on the extent to which they can access, use, and share scientific outputs, including genetic sequences, and, thereby, potentially challenge the adoption of open science at the continental level. As a result, there may be a need to harmonize the legislation and establish clarity, particularly with regard to the scientific research exceptions.

## V. CONCLUSION

The trend toward open science emerging in Africa and around the world is raising critical legal and ethical issues for research institutions, scientists, and other societal actors. Some of these issues, as shown in this paper, revolve around the underlying principles of intellectual property standards, which, in failing to capture the inclusive and collaborative nature of open science, could limit the extent to which open practices would be adopted in genomics health research and innovation in Africa. As a legal solution, this paper, among other things, proposes an inclusive intellectual property regime that allows the shared use and development of intellectual resources within the open science context.

## Footnotes

1 Purity Wambui Nyaikamba, Shingai Machingaidze, Gerald Chirinda, *The African Pharmaceutical Manufacturing Industry: Opportunities and challenges for growth*’, Future Africa Forum – Resilient Futures Nov. 28, 2022, https://forum.futureafrica.com/the-african-pharmaceutical-manufacturing-industry-opportunities-and-challenges-for-growth/ (accessed May 20, 2024).

2
*Id.*; Michael Conway, Tania Holt, Adam Sabow, and Irene Yuan Sun, *Should Sub-Saharan Africa Make its Own Drugs?*, Jan. 10, 2019, https://www.mckinsey.com/industries/public-sector/our-insights/should-sub-saharan-africa-make-its-own-drugs#/ (accessed May 20, 2024).

3 Nyaikamba, Machingaidze, & Chirinda, *supra* note 1.

4 David Cox, *The Search for the World’s ‘Missing’ Genomes*, BBC Future – Beyond the Genome, Feb. 28, 2023, https://www.bbc.com/future/article/20230227-the-search-for-the-worlds-missing-genomes (accessed May 20, 2024); S, Fatumo et al*.*, *Promoting the Genomic Revolution in Africa through the Nigerian 100 K Genome Project*, 54 Nat Genet, 531, 531–536 (2022), https://doi.org/10.1038/s41588-022-01071-6.

5 Olabode E. Omotoso et al., *Bridging the Genomic Data Gap in Africa: Implications for Global Disease Burdens*, 18(1) Globalization and Health, 1, 1–8, (2022), https://doi.org/10.1186/s12992-022-00898-2.

6 UNESCO, Recommendation on Open Science 2021, paras. 6–11.

7
*Id*. at Preamble.

8 UNESCO, *UNESCO Recommendation on Open Science*, https://www.unesco.org/en/open-science/about?hub=686 (accessed May 20, 2024).

9 For a more elaborate discussion of instruments such as ‘intellectual property policy’, ‘intellectual property strategy’, ‘intellectual property policy’, ‘intellectual property plan’, and ‘intellectual property development plan’ within the African context, see Caroline B. Ncube, Intellectual Property Law in Africa: Harmonising Administration and Policy 37–66 (2nd ed. Routledge 2023)

10
*Id*. at 53–54.

11
*Id*. at 54.

12
*Id.*

13
*Id*. at 40.

14 WIPO, *WIPO Lex*, https://www.wipo.int/wipolex/en/index.html (accessed May 24, 2024).

15 The Revised Bangui Agreement 2015, Art 5(2).

16
*Id.*

17 The Harare Protocol on Patents and Industrial Designs 1982 (as amended), Sec. 2(3).

18 See, the COMESA Policy on Intellectual Property Rights, 2013; The Protocol on the Establishment of the East African Community Common Market (CMP) 2010; EAC Regional Intellectual Property Policy on the Utilisation of Public Health-Related WTO-TRIPS Flexibilities and the Approximation of National Intellectual Property Legislation 2013; ECOWAS Harmonised TRIPS Policy 2012; The SADC Regional Framework and Guidelines on Intellectual Property 2018.

19 Juliet Ogbodo, *AfCFTA Phase II: Towards active participation of ECOWAS in the Intellectual Property Rights Negotiations*, Afronomicslaw, Oct. 30, 2019, https://www.afronomicslaw.org/2019/10/30/afcfta-phase-ii-towards-active-participation-of-ecowas-in-the-intellectual-property-rights-negotiations (accessed May 24, 2024).

20
Adejoke Oyewunmi, Nigerian Law of Intellectual Property 6–8, 23 (University of Lagos Press and Bookshop Ltd, Lagos, Nigeria 2015).

21 Donrich Thaldar, *The Wisdom of Claiming Ownership of Human Genomic Data: A Cautionary Tale for Research Institutions*, Developing World Bioethics, 1, 1–8 (2024), https://doi.org/10.1111/dewb.12443

22 Nigerian Patents and Designs Act 1970, Sec. 1(4); The Kenyan Industrial Property Act 2001, Sec. 21(3)(a); The South African Patents Act 1978, Sec. 24(2)(a); The Ghanaian Patents Act 2003, Sec. 2(a).

23 National Human Genome Research Institute (NHGRI), *Intellectual Property in Genomics* (2019), https://www.genome.gov/about-genomics/policy-issues/Intellectual-Property (accessed May 24, 2024).

24
*Id.*

25
*Id.*

26
*Id.* MedlinePlus, *Can Genes be Patented?*  https://medlineplus.gov/genetics/understanding/testing/genepatents/#:~:text=A%20gene%20patent%20is%20the,have%20first%20identified%20the%20gene (accessed May 24, 2024).

27 MedlinePlus, *supra* note 26.

28 NHGRI, *supra* note 23.

29 Aishatu E. Adaji, *Patenting in Biotechnology: An Analysis of the Three Tests of Patentability under the Nigerian Patent Law*, 11(1) Journal of Commercial and Property Law 24, 24–35 (2024), https://journals.unizik.edu.ng/jcpl/article/download/3571/2892 (accessed May 24, 2024).

30 569U.S. 576 (2013).

31 Thaldar, *supra* note 21, at 3.

32 The Kenyan Copyright Act 2001 (as amended), Sec. 2(1); the Nigerian Copyright Act 2022, Sec. 108; WIPO Copyright Treaty 1996, Art. 4; WTO TRIPS Agreement, Art. 10.1; Cf The South African Copyright Amendment Act 125 of 1992 and The Ghanaian Copyright Act 2005, Sec 1(1)(h).

33 The Kenyan Copyright Act 2001 (as amended), Sec. 26; the Nigerian Copyright Act 2022, Sec. 9; The South African Copyright Act 1978, Sec. 6; The Ghanaian Copyright Act 2005, Sec 5.

34 Jerome H. Reichman and Ruth L. Okediji, *When Copyright Law and Science Collide: Empowering Digitally Integrated Research Methods on a Global Scale* 96(4) *Minnesota Law Review* 1362, 1369 (2012); See also P.U.K De Silva and C.K Vance, Scientific Scholarly Communication: The Changing Landscape 17–23 (Springer International Publishing AG 2017).

35 Donrich W. Thaldar et al., *The Multidimensional Legal Nature of Personal Genomic Sequence Data: A*
*South African Perspective*, 13 Front. Genet. (2022).

36 R.S Eisenberg, *Proprietary Rights and the Norms of Science in Biotechnology Research*, 97 Yale Law Journal 177, 183 (1987); A.K Rai, *Regulating Scientific Research: Intellectual Property Rights and the Norms of Science*, 94 Northwestern University Law Review 77, 88 (1999).

37 UNESCO Recommendation on Open Science, https://www.unesco.org/en/open-science/about?hub=686 (accessed Jun 24, 2024).

38 D. Thaldar, A. Gooden & M. Steytler, *Open Science and Human Genetic Data: Recommendations on South Africa’s Draft National Open Science Policy*, Front. Genet. 14:1248747 (2023).

39 O. Oaiya, *National Open Science Policies in Africa Existing or in Preparation*, https://www.unesco.org/sites/default/files/medias/fichiers/2022/05/National_and_Institutional_OS_Policies.pdf (accessed Jun 24, 2024).

40 The Open COVID Pledge, *Make the Pledge to Share Your Intellectual Property in the Fight Against COVID-19*, https://opencovidpledge.org (accessed Jun. 24, 2024); Apache Software Foundation (ASF), *Licenses*, http://www.apache.org/licenses/ (accessed Jun. 24, 2024); Eben Moglen, *Why the FSF Gets Copyright Assignments from Contributors*, https://www.gnu.org/licenses/why-assign.en.html (accessed Jun. 24, 2024); CAMBIA, *The CAMBIA Bios Initiative*, https://cambia.org/bios-landing/the-cambia-bios-initiative/ (accessed Jun. 24, 2024).

41 The Model Open Science Policy of Nigeria 2024, para 3.1.ii.8.

42 Creative Commons, *About CC Licenses*, https://creativecommons.org/share-your-work/cclicenses/ (accessed Jun. 24, 2024); Open Source Initiative (OSI), *OSI Approved Licenses*, https://opensource.org/licenses (accessed Jun. 24, 2024); The Free Software Foundation, *Licenses*, https://www.gnu.org/licenses/licenses.html (accessed Jun. 24, 2024); ‘GNU’, as explained by Richard Stallman, is a recursive acronym that stands for “GNU's Not Unix!” - The Free Software Foundation, How To Pronounce GNU, https://www.gnu.org/gnu/pronunciation.html (accessed Nov 30, 2024).

43
Caroline B. Ncube, Science, Technology & Innovation and Intellectual Property: Leveraging Openness for Sustainable Development in Africa, 127 (Juta and Company Ltd, Cape Town 2021).

44 J. Bessen and E. Maskin, *Sequential Innovation, Patents, and Imitation*, 40 The Rand Journal of Economics 611, 613 (2009), https://doi.org/10.1111/j.1756-2171.2009.00081.x (accessed Jun 24, 2024).

45
de Silva and Vance, *supra* note 34 at 21.

46 I.E Anyira and I. Idubor, *Open Access Resources: Option for Libraries in a Challenged Economy*, Library Philosophy and Practice 4202 (2020), https://digitalcommons.unl.edu.libphilprac/4202, (accessed May 24, 2024) (emphasizing the challenge that ‘high cost of books, journals and database subscriptions’ pose to Nigeria and other African countries)

47 UNESCO, *supra* note 6 at Preamble, para 16b.

48 The Free Software Foundation, *What Is Copyleft*, https://www.gnu.org/licenses/copyleft.en.html (accessed Jun. 24, 2024).

49 Arti K. Rai, *Critical Commentary on ‘Open Source’ in the Life Sciences*, in Gene Patents and Collaborative Licensing Models: Patent Pools, Clearinghouses, Open Source Models and Liability Regimes 214–216 (Van Overwalle G. ed., 2009); Donrich Thaldar, *Open Science and Its Enemies: Policy Initiatives in South Africa* (2023), https://blog.petrieflom.law.harvard.edu/2023/10/10/open-science-and-its-enemies-policy-initiatives-in-south-africa/ (accessed Jun. 25, 2024).

50 Rai, *supra* note 49 at 214.

51 Alison Parker, *Open Science and Intellectual Property: Using Open Licenses to Open Your Science*, Wilson Centre, Blog Post, Jan. 27, 2021, https://www.wilsoncenter.org/blog-post/open-science-and-intellectual-property-using-open-licenses-open-your-science (Jun. 25, 2024).

52 Javier de la Cueva and Eva Méndez, *Open Science and Intellectual Property Rights: How can they better interact? State of the Art and Reflections*, 72 (Report of Study, European Commission, 2022).

53
V. Lindberg, Intellectual Property and Open Source: A Practical Guide to Protecting Code, 171 (O’Reilly Media, Inc., California, United States 2008).

54 Parker, *supra* note 51.

55 G. Van Overwalle, *Creating Universal and Sustainable Access to Plants and Seeds: The Role of Clearinghouses, Open Source Licenses, and Inclusive Patents*, in The Commons, Plant Breeding and Agricultural Research: Challenges for Food Security and Agrobiodiversity, 97 (F Girard and C Frison eds. 2018).

56 Janet Hope, *Open Source Genetics: A Conceptual Framework*, in Gene Patents and Collaborative Licensing Models: Patent Pools, Clearing Houses, Open Source Models and Liability Regimes, 179 (G. van Overwalle ed. 2009); See also van Overwalle, *supra* note 55 at 97.

57 Sunimal Mendis, *Public Open Collaborative Creation (POCC): A New Archetype of Authorship*, 22 J World Intellect Prop. 59, 64 (2019).

58
*Id*. at 59–75.

59
*Id.* at 66–70; OYEWUNMI, *supra* note 20 at 32–49.

60 European Commission and Africa IP SME Helpdesk, *IP Fiche: Senegal*, 28, https://intellectual-property-helpdesk.ec.europa.eu/system/files/2022-12/Africa_IP-Country-Fiche_Senegal.pdf (accessed Jun. 24, 2024).

61 Mendis, *supra* note 57 at 62–65.

62
*Id.* at 66–67.

63 A look at Article 5 of Annex VII to the Bangui Agreement 1977, appears to suggest a distinction between ‘collective works’ and ‘collections of works’ within the context of the Agreement.

64 The South African Copyright Act 1978, Sec. 1(1)(xlvi); The Ghanaian Copyright Act 2005, Sec. 76; The Kenyan Copyright Act 2001 (as amended), Sec. 2(1); The Nigerian Copyright Act 2022, Sec. 108.

65 The South African Copyright Act 1978, Sec. 1 (xxvii) (d) and (g); The Ghanaian Copyright Act 2005, Sec. 76; The Kenyan Copyright Act 2001 (as amended), Sec. 2(1); The Nigerian Copyright Act 2022, Sec. 108; WIPO Copyright Treaty 1996, Art. 5; WTO TRIPS Agreement, Art. 10.2; Annex VII to the Bangui Agreement 1977, Arts.1(vi) and 5.

66 The Senegalese Copyright and Related Rights Law 2008, Art. 23 (No. 2008–09).

67 The Ghanaian Copyright Act 2005, Sec. 76; The Kenyan Copyright Act 2001 (as amended), Sec. 2(1); The Nigerian Copyright Act 2022, Sec. 76; WIPO Copyright Treaty 1996, Art. 5; WTO TRIPS Agreement, Art. 10.2; Annex VII to the Bangui Agreement 1977, Art. 5.2; The South African copyright law is silent in this regard.

68 Mendis, *supra* note 57 at 69–70.

69 Mitchell Joblin, et al., *Hierarchical and Hybrid Organizational Structures in Open-source Software Projects: A Longitudinal Study*, 32(4) ACM Trans. Softw. Eng. Methodol. 1 (2023), https://doi.org/10.1145/3569949.

70 Mendis, *supra* note 57 at 70.

71 The Nigerian Copyright Act, Sec. 29(a) - (b) and 2(5); WIPO Copyright Treaty 1996, Art. 5.2; WTO TRIPS Agreement, Art. 10.2; Annex VII to the Bangui Agreement 1977, Art. 5.2.

72 Mendis, *supra* note 57 at 68.

73
Oyewunmi, *supra* note 20 at 71; See also Mendis, *supra* note 57 at 69 (similarly pointing out that under the copyright law of the UK, a copyright work must ensue from the ‘prosecution of a common design’ among co-authors in order to qualify as a joint work).

74 Mendis, *supra* note 57 at 69.

75 Benoit Demil and Xavier Lecocq, *Neither Market nor Hierarchy nor Network: The Emergence of Bazaar Governance*, 27(10) Organization Studies, 1447, 1455 (2006).

76
Oyewunmi, *supra* note 20 at 69.

77
*Id*.; See also Mendis, *supra* note 57 at 67–68 (particularly offering a similar interpretation of the copyright laws of selected jurisdictions, including the US and the UK).

78
Oyewunmi, *supra* note 20 at 69, 71–72. (citing the cases of *Donoghue v. Allied Newspapers Ltd* (1937) 13 All ER 503, where the court held that the plaintiff neither qualifies as a sole or joint author of an article, even though the article was based on the series of interviews, he granted about his racing career. Rather, the court recognizes the authorship of the person who, using his own language and skill, expressed the thoughts and materials provided by the plaintiff during the interviews; and *Tate v. Fullbrook* (1908) 1 KB 821, where it was similarly held that the contributor of general ideas on which a work is based does not qualify as a joint author).

79 Mendis, *supra* note 57 at 67–68.

80
*Id.*

81
*Id.* at 71.

82 The Ghanaian Patents Act 2003, Art. 4(2); The Kenyan Industrial Property Act 2001, Sec. 30(2); The South African Patents Act 1978, Sec. 27(2)

83 The Nigerian Patents and Designs Act 1970, Sec. 2(5); Similarly, section 2 of the Kenyan Industrial Property Act 2001 defines an inventor as the ‘person who actually devises the invention….’; There are subtle references in this regard under Sections 1 and 4 of the Ghanaian Patents Act 2003.

84
de Silva and Vance, *supra* note 34 at 7.

85
*Id.*; H.T Lie, C Grimpe and R Sorheim, *Access and Openness in Biotechnology Research Collaborations Between Universities and Industry*, 37 Nature Biotechnology 1413, 1413–1419 (2019), https://doi.org/10.1038/s41587-019-0324-7.

86 Van Overwalle, *supra* note 55 at 96–97; Although, certain open-source licenses, such as the Creative Commons licence CC0, are purposely designed to formalize the transfer of intellectual property rights to the public domain.—Severine Dusollier, *Inclusivity in Intellectual Property*, in Intellectual Property And General Legal Principles: Is Ip A Lex Specialis? 110 (G.B Dinwoodie, ed., Edward Elgar Publishing 2015).

87 Toshiko Takenaka, *Inclusive Patents for Open Innovation*, 29 Tex. Intell. Prop. L. J. 187, 193 (2020).

88
*Id.* at 199.

89 AfCFTA Protocol on IPRs, Art. 2.

90
*Id.*, Arts. 2.2.i and 21.

91
*Id.*, Art. 23c.

92 Dusollier, *supra* note 86; van Overwalle, *supra* note 55; Mendis, *supra* note 57; Takenaka, *supra* note 87.

93 Takenaka, *supra* note 87 at 222.

94
*Id.* at 223.

95
*Id.* at 192.

96
*Id.*

97 Mendis, *supra* note 57 at 70.

98 The Nigerian Copyrights Act 2022, Sec. 20(1)(r).

99
*Id.* Sec. 20(1)(n).

100 The South African Patents Act 1978, Secs. 55–56; the Ghanaian Patents Act 2003, Secs. 13–14; the Nigerian Patents and Designs Act 1970, Sec. 11 and Part 1 of the First Schedule; the Kenyan Industrial Property Act 2001, Sec. 58.

101 A.E Adaji and A.R Isa, *The Viability of Patent-Related Flexibilities in Promoting Biotechnology Research and Innovation for Improved Food Security and Public Health in Nigeria* 7(1) Runlawj (2024), https://www.runlawjournals.com/index.php/runlawj/article/view/79.

102
*Id.*

103
*Id.*; World Trade Organization, World Health Organization and World Intellectual Property Organization, Promoting Access to Medical Technologies and Innovation: Intersections Between Public Health, Intellectual Property And Trade 91–92 (Switzerland, 2020).

