# Correction to: Intellectual property issues for open science practices in genomic-related health research and innovation in Africa

**DOI:** 10.1093/jlb/lsaf013

**Published:** 2025-08-13

This is a correction to: Aishatu Eleojo Adaji, Lukman Adebisi Abdulrauf, Intellectual property issues for open science practices in genomic-related health research and innovation in Africa, *Journal of Law and the Biosciences*, Volume 11, Issue 2, July-December 2024, https://doi.org/10.1093/jlb/lsae026

The article has been updated online to acknowledge funding support from the US National Institute of Mental Health and the US National Institutes of Health under the Harnessing Data Science for Health Discovery and Innovation in Africa (DS-I Africa) program, and to clarify that the content of the article is solely the responsibility of the authors and does not necessarily represent the official view of the US National Institute of Medical Health and the US National Institutes of Health.

